# Integration of widely targeted and targeted metabolomics reveals flavonoid accumulation profiles across different cultivars of *Ludisia discolor*

**DOI:** 10.3389/fpls.2026.1881198

**Published:** 2026-07-07

**Authors:** Luan Li, Junjie Yang, Tianxiang Zhang, Kunxiu Cai, Fenfen Wang, Qi Yang, Xiuxiang Lin, Ying Chen, Tao Zheng

**Affiliations:** 1 Party and Government Office, Fujian Institute Of Tropical Crops, Zhangzhou, China; 2 Public Laboratory, Fujian Institute Of Tropical Crops, Zhangzhou, China; 3 Biotechnology Laboratory, Fujian Institute Of Tropical Crops, Zhangzhou, China; 4 Fruit and Vegetable Laboratory, Fujian Institute Of Tropical Crops, Zhangzhou, China; 5 College of Landscape Architecture and Art, Fujian Agriculture and Forestry University, Fuzhou, China

**Keywords:** cultivar differentiation, flavonoids, *Ludisia discolor*, quality evaluation, targeted quantification, widely targeted metabolomics

## Abstract

**Introduction:**

*Ludisia discolor*, a rare orchid species, possesses significant medicinal value; however, the metabolic heterogeneity among its cultivars and the specific accumulation patterns of bioactive flavonoids remain largely uncharacterized.

**Methods:**

We employed an integrated approach combining widely targeted and targeted metabolomics data to systematically elucidate the metabolic profile differences among five major *L. discolor* cultivars (Funongdanxia [DX], Funongruyi [RY], Xigonghong [XGH], Minreyinlong [YL], and Minreyuanshuai [YS]).

**Results:**

A total of 2,286 metabolites were identified, from which 802 differential metabolites were identified, with the majority belonging to flavonoids, lipids, and terpenoids. Principal Component Analysis (PCA) and Orthogonal Partial Least Squares Discriminant Analysis (OPLS-DA) revealed that the metabolic profiles of the XGH and YL cultivars were significantly distinct from the others. KEGG pathway enrichment analysis indicated that phenylpropanoid biosynthesis, flavonoid biosynthesis, and flavone and flavonol biosynthesis are the core pathways driving cultivar differentiation. Additionally, three common differential Kaempferol-4'-O-glucoside*, 1-O-(3,4,5-Trimethoxybenzoyl)-β-D-Glucopyranoside, and sec-o-Glucosylhamaudol were identified as potential key chemical markers for cultivar discrimination. Targeted quantitative analysis further validated that XGH and YL possess a significant advantage in the accumulation of 11 key flavonoid components, with flavonol glycosides exhibiting the most prominent accumulation characteristics.

**Discussion:**

This study represents the first system-level investigation into the metabolic diversity of *L. discolor* cultivars, uncovering the unique advantages of XGH and YL in flavonoid accumulation and their specific pathway regulatory modes. These findings provide a solid scientific foundation and data support for the metabolic characterization of medicinal germplasm resources, the breeding of superior high-flavonoid cultivars, and the precise quality evaluation of medicinal materials.

## 1 Introduction

*Ludisia discolor* (Ker Gawl.) A. Rich., commonly known as the Jewel Orchid or “Gongshisong”, is a perennial herbaceous plant within the Orchidaceae family (Yu et al., 2019). While primarily distributed across Fujian, Guangdong, Hainan, Guangxi, and Yunnan provinces in China, it is also indigenous to Myanmar, Vietnam, and Thailand (Dressler, 1993). Beyond its ornamental value, *L. discolor* possesses a rich history in traditional ethnomedicine, where it is utilized to moisten the lungs, invigorate the spleen, and calm the mind. Clinically, it has been applied to treat conditions ranging from pulmonary tuberculosis and hemoptysis to neurasthenia and anorexia (Ze-Hao and Xian-Juan, 2013). Due to habitat destruction and over-exploitation driven by its medicinal and commercial value, *L. discolor* is currently listed as a Class II protected plant in China. Modern pharmacological studies have corroborated its traditional uses, revealing that the plant is rich in bioactive secondary metabolites, particularly flavonoids, terpenoids, and alkaloids, which underpin its significant therapeutic potential (Wang et al., 2024; Gao et al., 2024). Its high medicinal value now has also driven the rapid development of a cultivation-based industry, underscoring its increasing pharmaceutical relevance and economic significance.

The medicinal quality and therapeutic efficacy of medicinal plants are intrinsically linked to the accumulation of secondary metabolites. These compounds, primarily classified into terpenoids, alkaloids, and phenolics, are evolutionary adaptations to environmental stress. Among these, flavonoids (a subclass of phenolics) are of particular interest; they not only govern critical biological processes such as pigmentation and stress signaling but also confer potent antioxidant, anti-inflammatory, and anticancer properties (Brunetti et al., 2018; Li et al., 2020; Zhu et al., 2019). Research on other medicinal orchids, such as *Dendrobium* and *Anoectochilus*, has demonstrated that metabolite profiles are highly plastic, varying significantly based on tissue type, symbiotic relationships, and cultivation environment (Zhang et al., 2020; Xue-Yuan et al., 2018; Sha et al., 2025). While recent studies on *L. discolor* have begun to explore the biosynthetic genes associated with flavonoids and the mechanisms of leaf coloration (Guo et al., 2023), there remains a critical gap in our understanding of how metabolic profiles vary across different germplasm resources. Specifically, a systematic comparison of secondary metabolite accumulation among distinct cultivars is lacking. As metabolites are the phenotypic end-products of gene expression (Rinschen et al., 2019), defining these chemical variances is essential for the selection of superior cultivars, precise quality control, and the development of high-value medicinal products.

The advancement of metabolomics technique has provided a unique opportunity to better understand the plant chemistry. Unlike traditional phytochemical methods that focus on isolating single compounds, metabolomics offers a holistic view, capable of simultaneously detecting and quantifying hundreds of metabolites to reveal cryptic phenotypic differences. Specifically, widely targeted metabolomics combines the broad coverage of non-targeted approaches with the sensitivity and precision of targeted assays (Ma and Qi, 2021). This high-throughput strategy is particularly crucial for *L. discolor*, where the subtle interplay between flavonoids and other pathway intermediates defines cultivar quality. By decoding these chemical nuances, metabolomics can identify specific biomarkers for cultivar discrimination and elucidate the regulatory logic of biosynthetic pathways (Lan et al., 2024; Li et al., 2020; Shen et al., 2023), providing the precise data needed to transform breeding from an art into a science.

To address the lack of systematic metabolic data for *L. discolor*, this study employs an integrated approach combining widely targeted metabolomics with targeted quantitative analysis to characterize the leaf metabolic profiles of five representative cultivars. The specific objectives of this study are: (1) to comprehensively identify and quantify the metabolome of these cultivars to construct a detailed metabolic map; (2) to utilize multivariate statistical analyses, including Principal Component Analysis (PCA) and Orthogonal Partial Least Squares Discriminant Analysis (OPLS-DA), to identify key differential metabolites driving cultivar divergence; (3) to specifically elucidate the accumulation patterns of flavonoids and map the key KEGG pathways responsible for these variations; and (4) to evaluate the potential medicinal quality of different cultivars based on their metabolic signatures. The findings of this study will provide a theoretical basis and robust data support for germplasm identification, the breeding of high-flavonoid cultivars, and the standardization of *L. discolor* production.

## 2 Materials and methods

### 2.1 Plant materials

**Figure 1:**
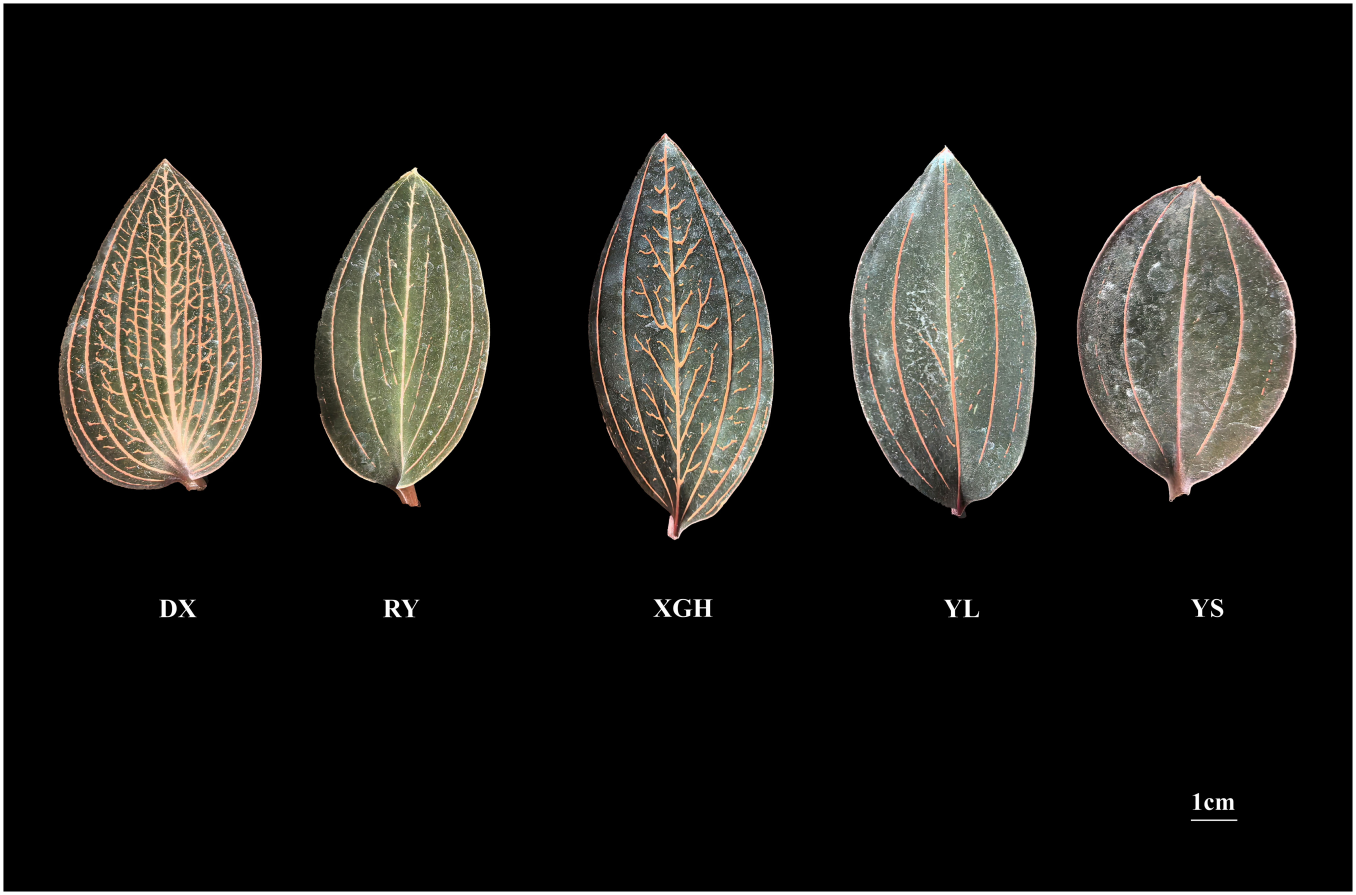
Leaf morphology of five *Ludisia discolor* cultivars. From left to right in the image are ‘DX’, ‘RY’, ‘XGH’, ‘YL’, and ‘YS’.

*Ludisia discolor* plants were obtained from the greenhouse of the South Subtropical Crop Germplasm Nursery Base, Fujian Institute of Tropical Crops, located at 24°38’N, 117°31’E. Five cultivars were selected for this study: ‘Xigonghong’, ‘Minre Yinlong’, ‘Minre Yuanshuai’, ‘Funong Danxia’, and ‘Funong Ruyi’. Among them, ‘Xigonghong’ is the major cultivated cultivar, whereas the other four are selected cultivars. These cultivars exhibit marked phenotypic differences in leaf shape, color, and venation patterns (**Figure 1**), which provided the basis for the comparative metabolomic analysis in this study. Functional leaves were collected from five healthy, uniformly growing six-month-old cultivars. For each group, leaves were collected from 10 plants, with 3–5 leaves sampled from each plant and pooled. The samples were wrapped in aluminum foil, immediately frozen in liquid nitrogen, and stored at −80 °C until analysis. Three biological replicates were prepared for each group.

### 2.2 Sample preparation and extraction for widely targeted metabolomics

Leaf tissues of *Ludisia discolor* were first subjected to vacuum freeze-drying for 63 h using a Scientz-100F lyophilizer. The dehydrated material was then pulverized into a fine, homogeneous powder with a mixer mill (MM 400, Retsch) operating at 30 Hz for 1.5 min. An accurately weighed 30 mg aliquot of the powder was extracted with 1.5 mL of pre-chilled 70% methanol in water supplemented with an internal standard. The suspension was thoroughly homogenized by vortex mixing (six cycles of 30 s each) and subsequently centrifuged at 12,000 rpm for 3 min at 4 °C. The clarified supernatant was carefully recovered, passed through a 0.22 μm membrane filter, and transferred into autosampler vials prior to UPLC–MS/MS analysis.

### 2.3 UPLC-ESI-MS/MS analysis

UPLC–ESI–MS/MS profiling was carried out using an ultra-performance liquid chromatography system coupled with tandem mass spectrometry (ExionLC™ AD, https://sciex.com.cn/). Chromatographic separation was achieved on an Agilent SB-C18 column (2.1 × 100 mm, 1.8 µm), with the column temperature maintained at 40 °C. The mobile phase consisted of ultrapure water containing 0.1% formic acid (solvent A) and acetonitrile supplemented with 0.1% formic acid (solvent B), delivered at a flow rate of 0.35 mL/min. Elution was performed using a programmed gradient: solvent B was held at 5% initially, increased linearly to 95% over 9.0 min, and maintained for 1 min before being reduced to 5% between 10.0 and 11.1 min, followed by re-equilibration until 14.0 min. The injection volume was set to 2 µL.

Mass spectrometric detection employed an electrospray ionization (ESI) source operating in both positive and negative ionization modes, with ion spray voltages of 5500 V and −4500 V, respectively. The source temperature was configured at 500 °C. Nitrogen was used as the nebulizing and auxiliary gases, with gas I at 50 psi, gas II at 60 psi, and curtain gas at 25 psi. Collision-induced dissociation was performed under high-energy conditions. Data acquisition was conducted in multiple reaction monitoring (MRM) mode; declustering potentials and collision energies were individually optimized for each ion transition during method development, and metabolite-specific MRM transitions were monitored according to their characteristic retention times during analysis.

### 2.4 Qualitative and quantitative analysis of metabolites

Metabolites were qualitatively identified using the Metware Database (Wuhan Metware Biotechnology Co., Ltd.) and public metabolite databases. Quantitative analysis was conducted using the MRM mode MSI Level 2. Peak area data for corresponding metabolites across different samples were integrated and corrected to determine relative metabolite content.

### 2.5 Quality control and differential metabolite analysis

Analytical reproducibility was assessed using a pooled quality control (QC) sample generated by combining equal aliquots of all *Ludisia discolor* leaf extracts. The QC samples were periodically injected after every six experimental samples, resulting in four QC data points, to monitor instrumental stability throughout data acquisition. This QC sample was subjected to the same preparation and instrumental procedures as the study samples to ensure consistency throughout the analytical workflow. Raw mass spectrometry data were processed using Analyst 1.6.3. A stable isotope-labeled internal standard-based normalization method was then applied to construct the standardized data matrix. Before sample extraction, a mixture of 13C/15N isotope-labeled internal standards at known concentrations, covering multiple metabolite classes, was added to each sample. During data preprocessing, the peak area of each metabolite feature was normalized to the peak area of the structurally closest isotope-labeled internal standard. This approach corrected systematic variation introduced during sample preparation, injection, and instrumental response, thereby generating normalized relative quantitative data. Principal component analysis (PCA) was first applied to visualize overall metabolic patterns and detect features contributing to sample differentiation. To enhance the identification of variables associated with group separation, orthogonal partial least squares–discriminant analysis (OPLS-DA) was subsequently performed, allowing discrimination-related variation to be distinguished from orthogonal, non-informative variability. The variable importance in projection (VIP) values were calculated using the orthogonal partial least squares discriminant analysis (OPLS-DA) model. Differential metabolites were identified based on VIP values, fold change (FC), and Benjamini-Hochberg FDR-corrected significance levels. Metabolites with VIP ≥ 1, FDR < 0.05, and |log_2_FC| ≥ 1 were considered significantly differential. Identified differential metabolites were further annotated against the Kyoto Encyclopedia of Genes and Genomes (KEGG) database, followed by pathway enrichment analysis to facilitate biological interpretation.

### 2.6 Sample preparation and extraction for quantitative flavonoid analysis

Leaf samples of *Ludisia discolor* designated for flavonoid quantification were first subjected to vacuum freeze-drying. The dried material was then ground into a fine powder using a mixer mill operating at 30 Hz for 1.5 min. A 20 mg portion of the powder was accurately weighed and extracted with 500 μL of pre-cooled 70% aqueous methanol containing an internal standard, with extraction facilitated by ultrasonication for 30 min. Absolute quantification of the targeted flavonoid compounds was performed using a combined internal standard and external calibration approach. For each target flavonoid, a corresponding reference standard was used to construct an external calibration curve. The chromatographic peak response of each target compound in the sample, after internal standard correction, was then applied to its respective external calibration curve to calculate the absolute concentration in the sample, thereby achieving compound-specific absolute quantification. The mixture was subsequently centrifuged at 12,000 r/min for 5 min at 4 °C to achieve phase separation. The resulting supernatant was carefully collected, filtered through a 0.22 μm membrane, and transferred into autosampler vials prior to LC–MS/MS analysis. Pathway enrichment analysis was performed using MetaboAnalyst 5.0 (Pang et al., 2021). The built-in pathway database integrates KEGG standard pathways as well as more specific functional sub pathways curated from literature and metabolite annotations. Therefore, the enrichment results may include both full KEGG pathways and metabolite-level subpathway entries. In this study, both specific subpathways and their related upstream pathways were considered in the biological interpretation.

### 2.7 Flavonoid quantification via UPLC-ESI-MS/MS

Flavonoid quantification was performed using an ultra-performance liquid chromatography–electrospray ionization tandem mass spectrometry (UPLC–ESI–MS/MS) platform integrating high-resolution chromatographic separation with sensitive mass detection (ExionLC™ AD, https://sciex.com.cn/; QTRAP^®^ 6500+, https://sciex.com.cn/). Metabolites were separated on a Waters ACQUITY UPLC HSS T3 C18 column. The mobile phases consisted of ultrapure water containing 0.05% formic acid (solvent A) and acetonitrile supplemented with 0.05% formic acid (solvent B).

Elution was carried out using a programmed gradient as follows: the initial A/B ratio was 90:10 at 0 min, adjusted to 80:20 at 1 min, and further shifted to 30:70 by 9 min. The composition reached 5:95 at 12.5 min and was maintained until 13.5 min, after which it was rapidly returned to 90:10 at 13.6 min and equilibrated until 16 min. The flow rate was maintained at 0.35 mL/min, the column temperature was set to 40 °C, and the injection volume was 2 μL.

Mass spectrometric detection was conducted using an electrospray ionization (ESI) source with a source temperature of 550 °C. Ion spray voltages were configured at 5500 V in positive mode and −4500 V in negative mode, with the curtain gas pressure set to 35 psi. Data acquisition on a QTRAP 6500+ mass spectrometer was performed using optimized declustering potential (DP) and collision energy (CE) parameters for each ion transition to ensure reliable and sensitive detection.

### 2.8 Qualitative and quantitative analysis of flavonoids

To evaluate the reproducibility and precision of the full workflow from sample extraction to instrumental analysis, three QC samples were independently prepared, including independent weighing, extraction, and injection, and analyzed in the same batch as the experimental samples. Qualitative analysis was performed using the MWDB (Metware Database) constructed based on authentic standards MSI Level 1. Upon obtaining mass spectrometry data, chromatographic peaks for all targets were integrated, and quantitative analysis was performed using standard curves. Data quality control was conducted as described in Section 2.5. Metabolites with VIP ≥ 1, Benjamini-Hochberg FDR < 0.05, and |log_2_FC| ≥ 1 were considered significantly differential.

## 3 Results

### 3.1 Quality assessment of widely targeted metabolomics data

**Figure 2:**
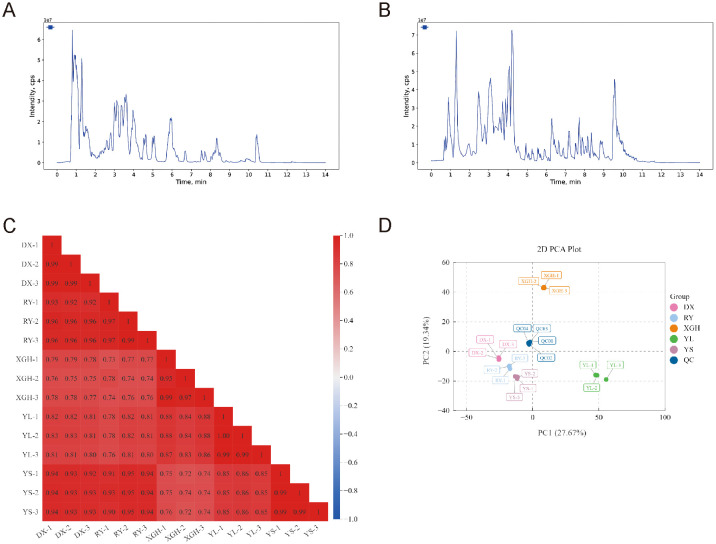
Quality assessment of leaf metabolome data from five *Ludisia discolor* cultivars. **(A)** Overlaid total ion chromatograms (TICs) of QC samples in positive ion mode. **(B)** Overlaid total ion chromatograms (TICs) of QC samples in negative ion mode. The high degree of overlap among all QC samples in terms of retention time and signal intensity demonstrates excellent stability and reproducibility of the LC-MS system throughout the data acquisition process. **(C)** Inter-sample correlation analysis. Numbers within the squares represent correlation coefficients between samples, with darker red indicating stronger positive correlation. **(D)** Principal component analysis (PCA) score plot of samples. PC 1 is the first principal component, and PC 2 is the second principal component; percentages indicate the explanatory power of each component for the overall dataset. Each point represents an individual replicate sample, with samples of the same cultivar shown in the same color.

QC analysis was conducted on the metabolomic data derived from the leaves of the different *L. discolor* cultivars. In both positive and negative ion modes, the total ion current (TIC) curves of QC samples exhibited consistent retention times and peak intensities with high overlap, indicating stable instrument signals and high data reliability and reproducibility throughout the experimental period (**Figures 2A, B**). Pearson correlation analysis revealed intra-group correlation coefficients greater than 0.9, confirming excellent reproducibility among biological replicates. Conversely, lower correlation coefficients were observed between the XGH/YL group and the DX/RY/YS group, confirming extensive differences in metabolite accumulation among cultivars (**Figure 2C**). PCA showed that PC1 and PC2 explained 27.67% and 19.34% of the total variance, respectively. The five cultivars exhibited clear intra-group clustering and inter-group separation trends (**Figure 2D**), with YL and XGH displaying particularly distinct separation from the other cultivars. Additionally, the tight clustering of the three QC samples further validated the instrumental stability and the high reliability of the acquired data (**Figure 2D**).

**Figure 3:**
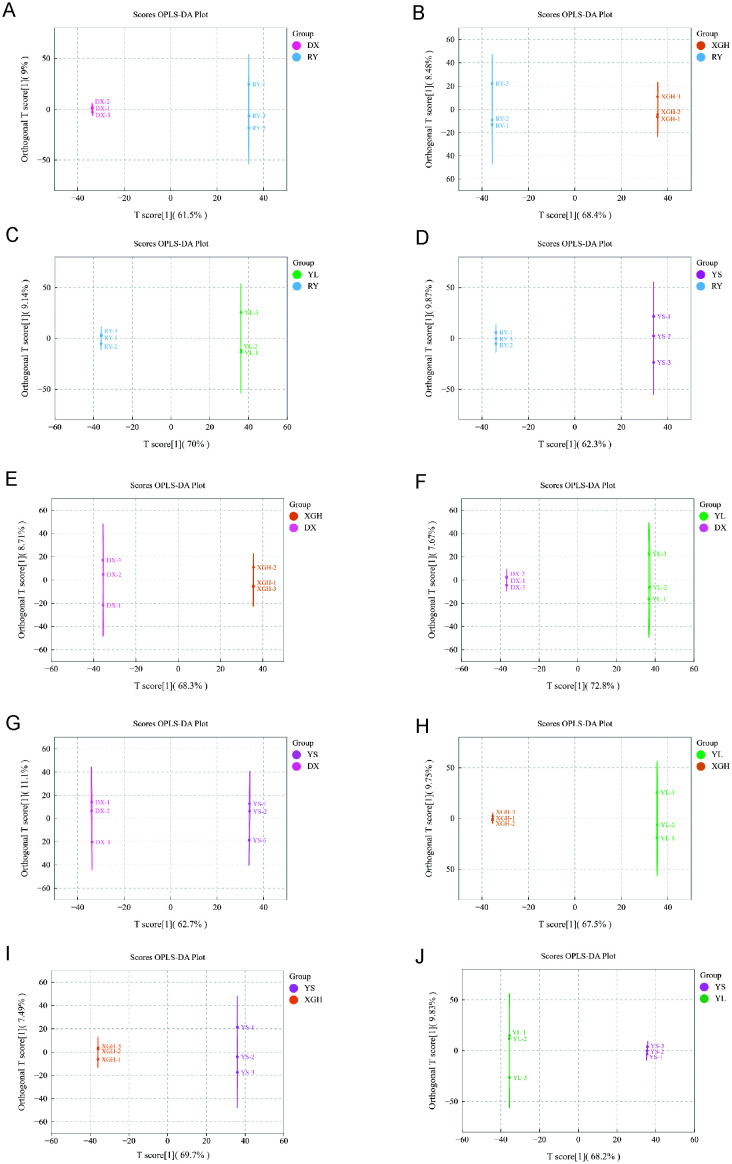
OPLS-DA score plots for each comparative group. **(A)** DX vs. RY; **(B)** XGH vs. RY; **(C)** YL vs. RY; **(D)** YS vs. RY; **(E)** XGH vs. DX; **(F)** YL vs. DX; **(G)** YS vs. DX; **(H)** YL vs. XGH; **(I)** YS vs. XGH; **(J)** YS vs. YL. In each subplot, the horizontal axis represents the predictive principal component T[1], and the vertical axis represents the orthogonal component T[1]orth, with the numbers indicating the explained variance. Data points in the plots represent individual samples, with colors corresponding to different cultivars. All comparative groups demonstrate good intra-group clustering and clear inter-group separation.

**Figure 4:**
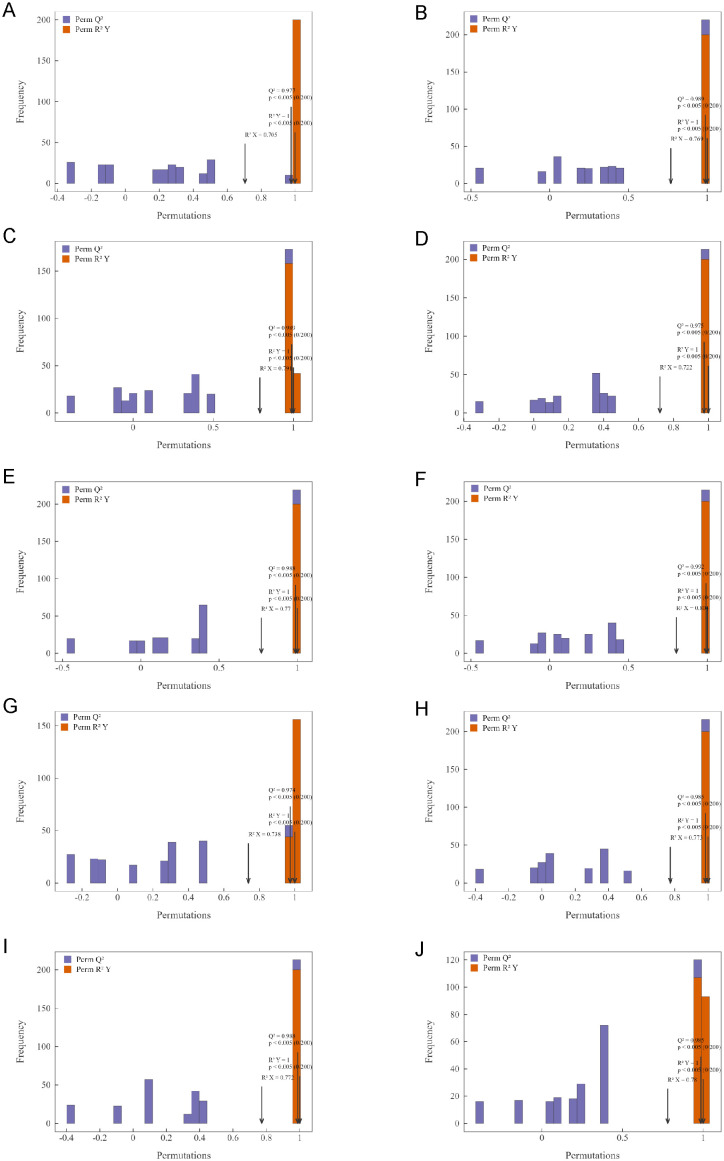
OPLS-DA model validation plots for each comparative group. **(A)** DX vs. RY; **(B)** XGH vs. RY; **(C)** YL vs. RY; **(D)** YS vs. RY; **(E)** XGH vs. DX; **(F)** YL vs. DX; **(G)** YS vs. DX; **(H)** YL vs. XGH; **(I)** YS vs. XGH; **(J)** YS vs. YL. The horizontal axis represents the permutation retention rate, and the vertical axis represents the intercept values of R²Y (the explained variance of the Y matrix, orange) and Q² (the model’s predictive ability, blue). Arrows indicate the statistical values of the original model. In all subplots, the ordinate of the intersection point between the regression line and the Q² axis (Q² intercept) is less than zero, and the original model’s R²Y and Q² values are significantly higher than those of the permutation distribution (*P* < 0.05). This confirms that the models are valid, carry no risk of overfitting, and are suitable for subsequent analysis.

To further investigate metabolic distinctions among cultivars, OPLS-DA were performed and results demonstrated clear separation for all pairwise comparisons. Notably, models comparing XGH or YL against other cultivars showed significant separation, with distinct differences in the predicted principal component T score [1], strongly corroborating the PCA findings (**Figure 3**). The model quality was evaluated using R^^2^X, R^^2^Y, and Q^^2^ parameters, where R^^2^Y represents the goodness of fit and Q^^2^ represents the predictive ability. Values closer to 1 indicate a more stable and reliable model (Q^^2^ > 0.5 indicates validity; Q^^2^ > 0.9 indicates excellence). Permutation tests (200 iterations) for the 10 pairwise comparison models yielded R^^2^X, R^^2^Y, and Q^^2^ values all exceeding 0.5, with R^^2^Y = 1 and Q^^2^ > 0.9 (**Figure 4**). This indicates that the established OPLS-DA models are robust, reliable, and free from overfitting, providing high confidence in the identified inter-cultivar differences.

### 3.2 Screening and identification of differential metabolites

**Figure 5:**
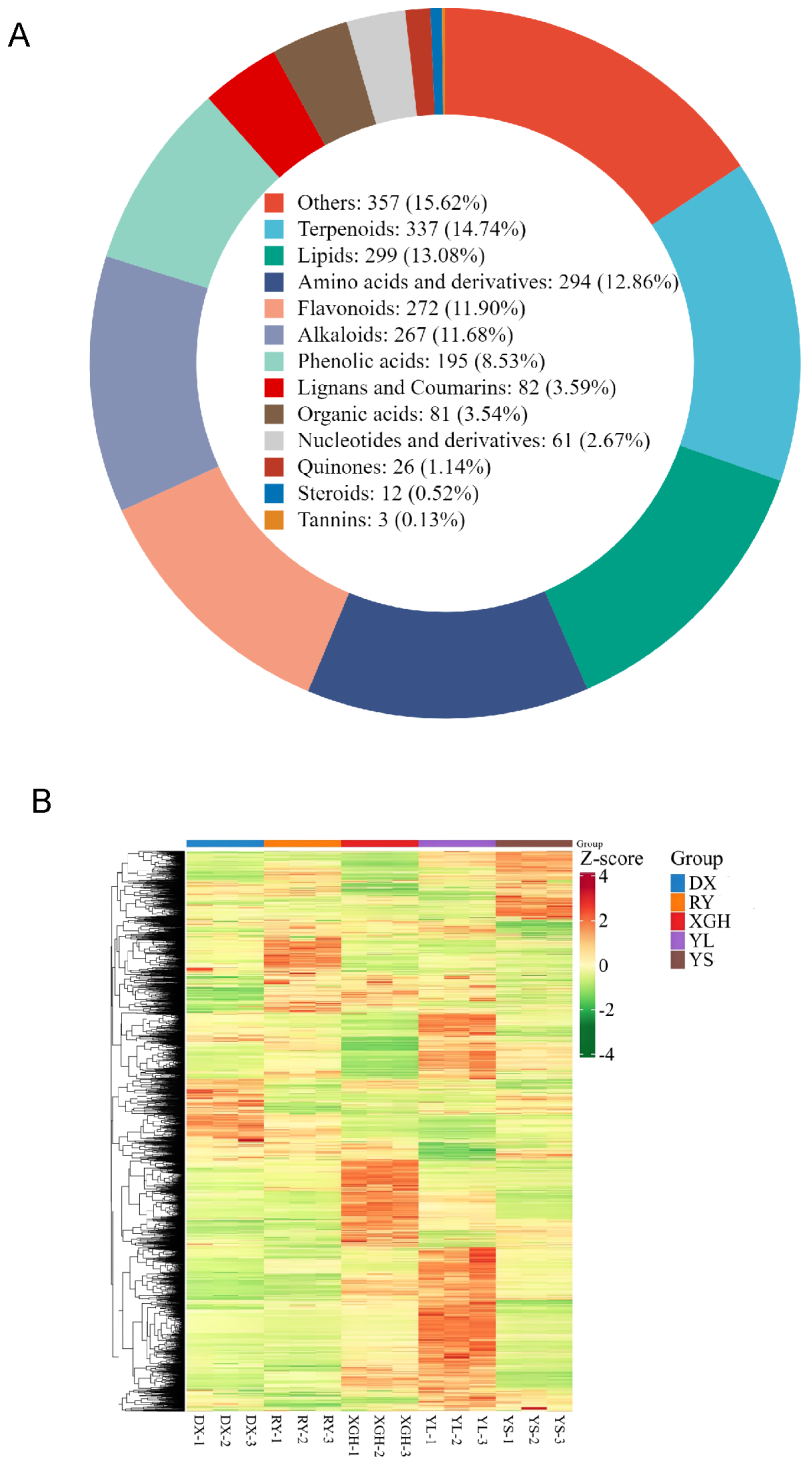
Overview of the identified metabolites. **(A)** Donut plot of metabolite classification. **(B)** Clustered heatmap based on the accumulation levels of all identified metabolites. The color gradient ranges from blue (negative Z-score, indicating abundance below the mean) to red (positive Z-score, indicating abundance above the mean).

A total of 2,286 metabolites were identified in the leaves of the five *L. discolor* cultivars, comprising 13 major categories. The predominant classes included 337 terpenoids (14.74%), 299 lipids (13.08%), 294 amino acids and derivatives (12.86%), 272 flavonoids (11.90%), and 267 alkaloids (11.68%), which collectively accounted for over half (64.26%) of the identified compounds (**Figure 5A**). Hierarchical cluster analysis revealed substantial metabolic divergence among the five cultivars, with DX, RY, XGH, YL, and YS forming distinct clusters. Notably, the metabolite accumulation patterns of XGH and YL were nearly opposite to those of the other three cultivars (**Figure 5B**).

**Figure 6:**
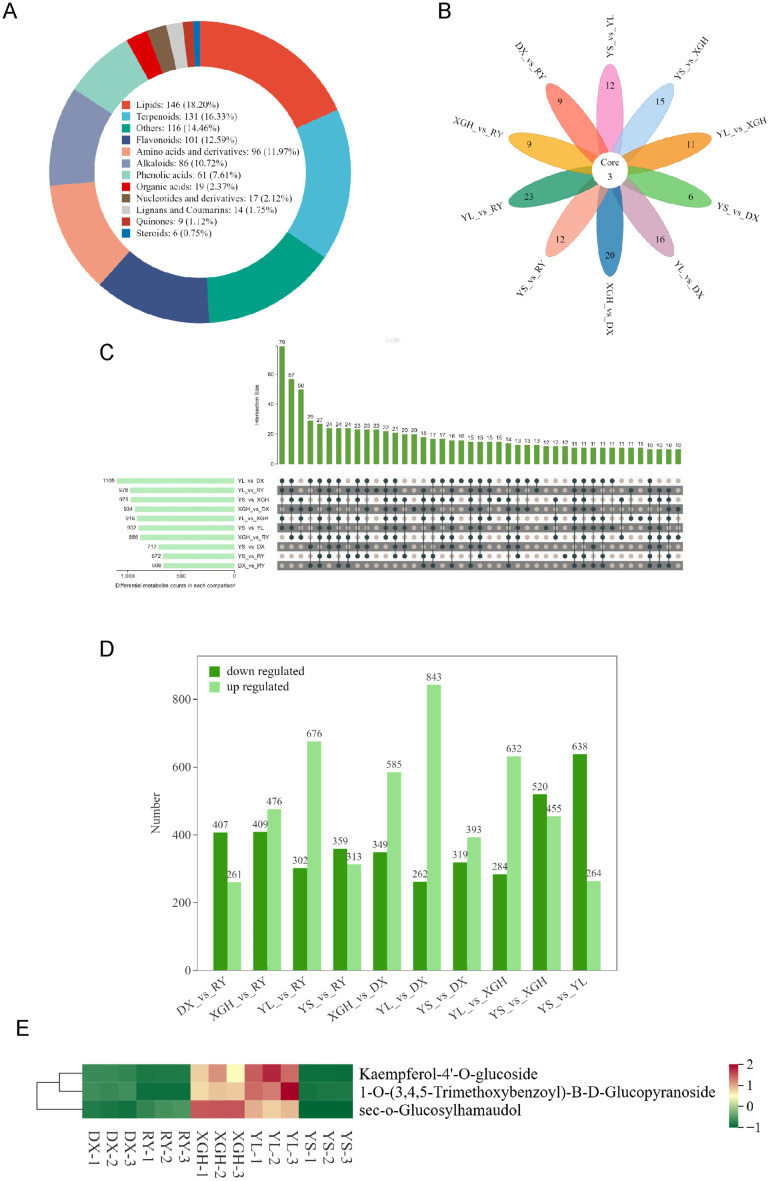
Overview of differential metabolites. **(A)** Donut plot of differential metabolite classification across the five cultivars. **(B)** Flower plot showing the number of differential metabolites for each comparative group. **(C)** UpSet plot displaying intersections of differential metabolite sets among all pairwise comparisons. This matrix-based visualization precisely illustrates set overlaps; the top bar chart indicates the number of metabolites in each intersection (defined by connected solid dots in the matrix below), while the left bar chart shows the total number of differential metabolites per comparison group. **(D)** Stacked bar chart for the numbers of up-regulated and down-regulated differential metabolites in each comparative group. **(E)** Clustering heatmap of three common differential metabolites across the five *Ludisia discolor* cultivars. The data are based on relative quantitative values obtained from widely targeted metabolomics. Each row represents one metabolite, and each column represents one biological replicate sample (n = 3). Relative abundance values were standardized using row-wise Z-score transformation and displayed using hierarchical clustering based on Euclidean distance. Color intensity represents relative abundance, ranging from −1 (green) to 2 (red). Clear clustering patterns were observed among sample groups, indicating cultivar-specific accumulation characteristics of these compounds. All differential metabolites met the following criteria: VIP ≥ 1 in the OPLS-DA model, |log_2_FC| ≥ 1, and Benjamini-Hochberg FDR < 0.05.

Differential metabolites were identified by combining multivariate statistical analysis (OPLS-DA VIP > 1) with univariate statistics (*p* < 0.05, |log_2_FC| ≥ 1, and FDR < 0.05.). A total of 802 differential metabolites were screened across the five cultivars, primarily consisting of lipids, terpenoids, and flavonoids (**Figure 6A**). Venn diagrams (**Figures 6B, C**) and bar charts (**Figure 6D**) illustrate the distribution of differential metabolites across comparison groups (detailed classification and counts are provided in **Supplementary Table 1**). The number of shared differential metabolites in specific comparisons was as follows: YL vs. RY (23), XGH vs. DX (20), YL vs. DX (16), YS vs. XGH (15), YS vs. YL (12), YS vs. RY (12), YL vs. XGH (11), DX vs. RY (9), XGH vs. RY (9), and YS vs. DX (6) (**Figure 6B**). Three differential metabolites were identified across all comparison groups, including Kaempferol-4’-O-glucoside*, 1-O-(3,4,5-Trimethoxybenzoyl)-β-D-Glucopyranoside, and sec-o-Glucosylhamaudol, which belonging to flavonols, phenolic acids, and chromones, respectively (**Figure 6B**). The accumulation patterns of the three common differential metabolites across cultivars are shown in **Figure 6E**. Kaempferol-4'-O-glucoside* and 1-O-(3,4,5-Trimethoxybenzoyl)-β-D-Glucopyranoside showed higher relative accumulation levels in the YL and XGH cultivars, whereas their levels were lower in DX, RY, and YS. The accumulation pattern of sec-o-glucosylhamaudol was slightly different, with the highest level observed in XGH and the lowest in YS. These results indicate that the three metabolites did not change uniformly across cultivars; instead, their accumulation patterns were closely associated with the distinctive metabolic phenotypes of YL and XGH.

**Figure 7:**
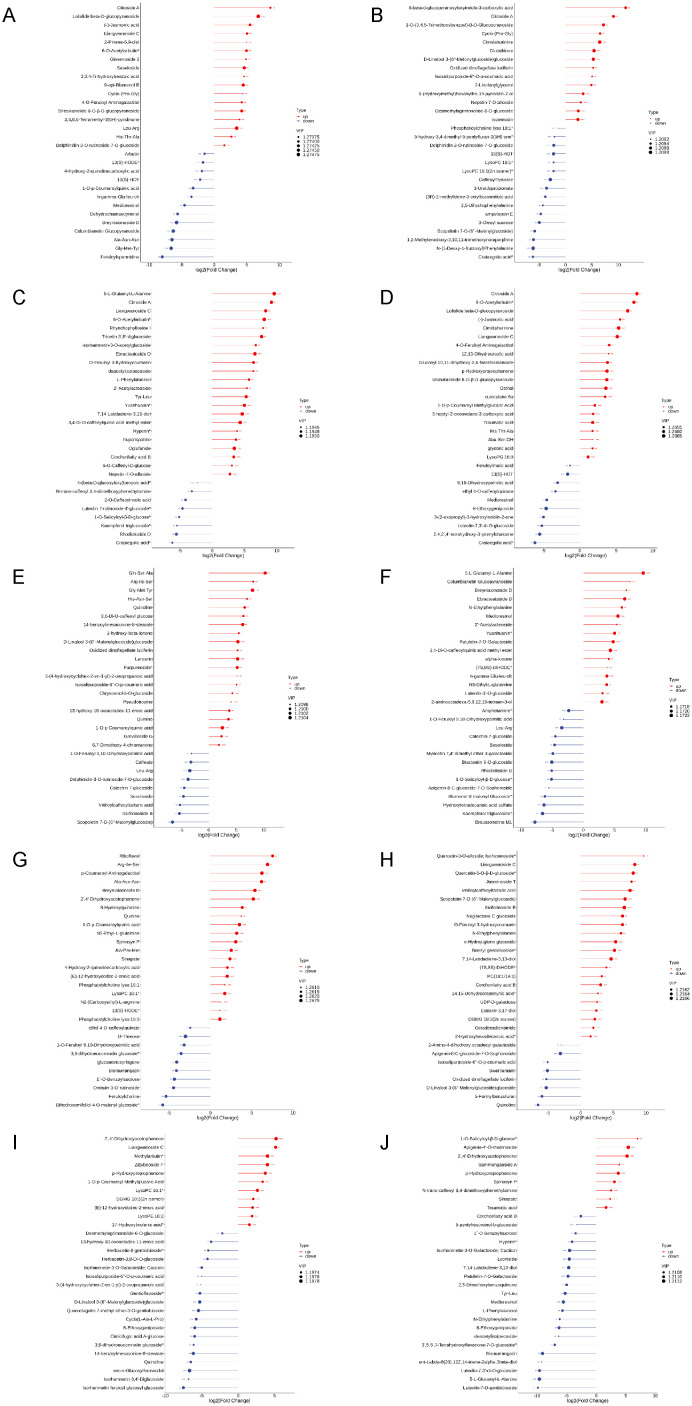
Lollipop plots of the top 30 differential metabolites ranked by VIP and |log2FC| in each comparison group. **(A)** DX vs. RY; **(B)** XGH vs. RY; **(C)** YL vs. RY; **(D)** YS vs. RY; **(E)** XGH vs. DX; **(F)** YL vs. DX; **(G)** YS vs. DX; **(H)** YL vs. XGH; **(I)** YS vs. XGH; **(J)** YS vs. YL. Each point represents a differential metabolite identified using the following criteria: VIP ≥ 1, |log_2_FC| ≥ 1, and FDR < 0.05. The length of each segment represents the |log_2_FC| value, and the circle size represents the VIP value.

To identify cultivar-specific metabolic signatures, lollipop charts were generated to visualize significant differential metabolites with top-ranking VIP and FC values for each comparison (**Figure 7**). Specifically, we examined pairwise comparisons, focusing first on XGH and YL due to their distinct metabolic profiles. In the comparison between XGH and RY, 885 differential metabolites were identified, with 476 up-regulated in XGH. These up-regulated compounds were dominated by flavonoids, amino acids, and terpenoids, including Glutathione, Kaempferol-4’-O-glucoside*, Citroside A, and D-Linalool 3-(6’’-Malonylglucoside) glucoside. Conversely, RY exhibited higher levels of compounds such as CaffeoylTyrosine, 13(S)-HOT, and Delphinidin-3-O-rutinoside-7-O-glucoside (**Figure 7B**). Similarly, the YL vs. RY comparison yielded 978 differential metabolites. YL was characterized by the up-regulation of 676 metabolites, including numerous flavonoids like Kaempferol-4’-O-glucoside*, Tricetin 3’,5’-diglucoside, and Yuanhuanin*, as well as terpenoids such as 7,14-Labdadiene-3,13-diol. In contrast, RY was enriched in N-trans-caffeoyl 3,4-dimethoxyphenethylamine and Luteolin 7-rutinoside-4’-glucoside* (**Figure 7C**).

Among the examined comparisons, the divergence between YL and DX was the most pronounced, yielding 1,105 differential metabolites, 843 of which were up-regulated in YL. This upregulation was mainly driven by flavonoids such as Patuletin-7-O-Galactoside and Yuanhuanin* alongside other metabolites like Medioresinol and alpha-Ionone. DX, however, retained higher levels of specific flavonoids like Bracteatin 6-O-glucoside and Apigenin-8-C-glucoside-7-O-Sophoroside (**Figure 7F**). When comparing XGH to DX, XGH displayed significant up-regulation of 585 metabolites, including five specific flavonoids (e.g., Lancerin, Chrysoeriol-5-O-glucoside) and four alkaloids (e.g., Quinine, Pseudotropine), whereas DX was enriched in phenolic compounds such as Caffeate and Vanilloylcaffeoyltartaric acid (**Figure 7E**).

In terms of the comparison between YS and YL, 902 differential metabolites were identified, with the majority (638) down-regulated in YS. YL exhibited significantly higher accumulation of flavonoids, amino acid derivatives, and terpenoids, such as 5-L-Glutamyl-L-Alanine and Luteolin-7,3’-di-O-glucoside, while YS showed higher expression of Apigenin-4’-O-rhamnoside and Spinosyn P (**Figure 7J**). Similarly, in the comparison between YS and XGH, XGH showed up-regulation of 520 metabolites relative to YS. Notably, eight significantly differential flavonoids were all down-regulated in YS, while XGH was enriched in compounds like Gentioflavoside* and Cimicifugic acid A-glucose (**Figure 7I**). The comparison between YL and XGH revealed 916 differential metabolites, with YL showing higher levels of terpenoids, lipids, and flavonoids such as Ilicifolinoside B and Quercetin-5-O-β-D-glucoside*, whereas XGH was enriched in Swertiamarin and Oxidized dinoflagellate luciferin (**Figure 7H**).

The metabolic differences were less pronounced in comparisons involving RY and DX, with 668 differential metabolites identified. Notably, DX was enriched in terpenoids like Loliolide beta-D-glucopyranoside and Ginsenoside 2, while RY showed lower levels of Leu-Arg and (-)-Jasmonic acid (**Figure 7A**). Similarly, the comparison between YS and RY revealed primarily terpenoid-driven differences, with YS accumulating Liangwanoside C and Traumatic acid, while RY was enriched in 6-Ethoxygeniposide and Luteolin-7,3’-di-O-glucoside (**Figure 7D**). Regarding the comparison between YS and DX, alkaloids emerged as the primary differentiators and YS was enriched in quinoline alkaloids like 8-Hydroxyquinoline, whereas DX contained higher levels of isoquinoline alkaloids. Notably, three key flavonoids were significantly down-regulated in YS compared to DX (**Figure 7G**).

In summary, XGH and YL are distinguished by marked flavonoid accumulation compared to RY, with YL exhibiting the most distinct flavonoid regulation. YS is generally characterized by lower flavonoid content but distinct alkaloid profiles compared to DX.

### 3.3 KEGG annotation and enrichment analysis of differential metabolites

**Figure 8:**
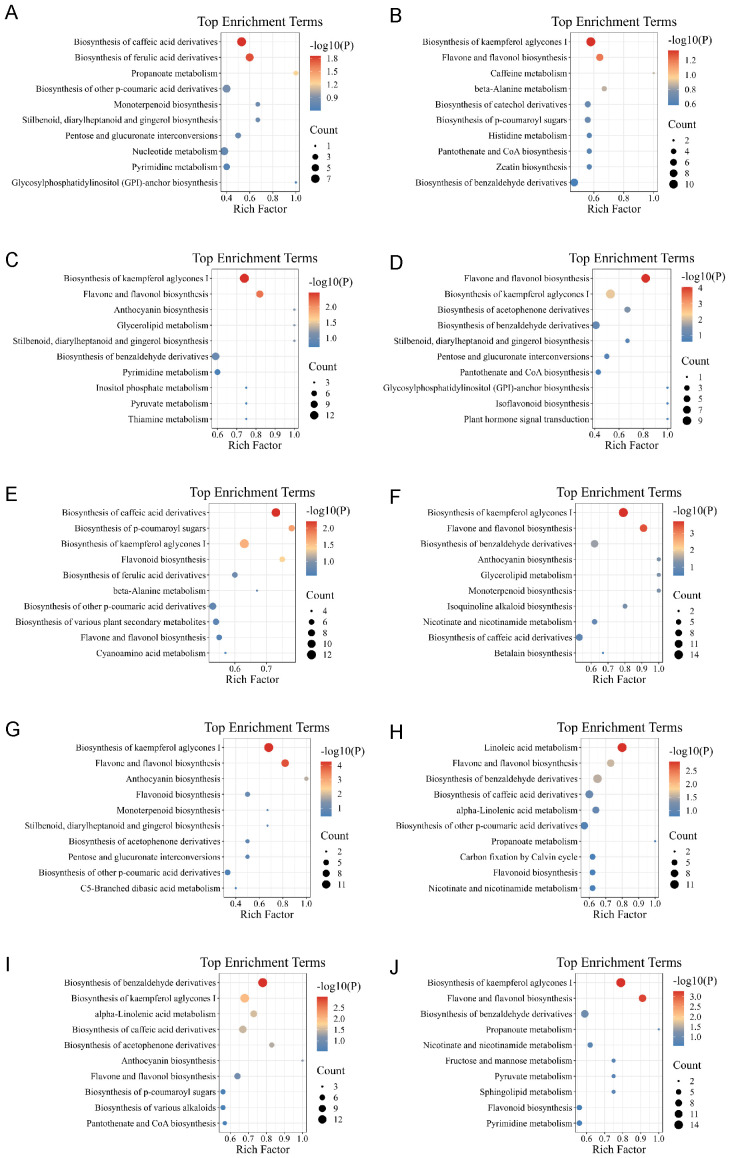
KEGG enrichment analysis of differential metabolites for each comparative group. **(A)** DX vs. RY; **(B)** XGH vs. RY; **(C)** YL vs. RY; **(D)** YS vs. RY; **(E)** XGH vs. DX; **(F)** YL vs. DX; **(G)** YS vs. DX; **(H)** YL vs. XGH; **(I)** YS vs. XGH; **(J)** YS vs. YL. Pathway enrichment analysis was performed using MetaboAnalyst 5.0. The top 10 metabolic pathways significantly enriched (*P* < 0.05) with differential metabolites are displayed. Each bubble represents a pathway; bubble size corresponds to the number of differential metabolites mapped to that pathway, and bubble color represents the significance level of enrichment (-log_10_(*P*-value), gradient from blue (not significant) to red (highly significant)). The x-axis (Enrichment Factor) is the ratio of the number of differential metabolites mapped to a pathway to the total number of metabolites annotated to that pathway; a higher value indicates a greater degree of enrichment.

To interpret the biological significance of the observed metabolic variations, differential metabolites were mapped to the KEGG database for pathway enrichment analysis. This analysis successfully identified metabolic pathways closely associated with the differential expression patterns, providing a foundation for functional gene mining. The number of enriched pathways varied by comparison group, ranging from 61 in YS vs. RY to 92 in YL vs. RY. The top 10 pathways ranked by P-value were visualized to highlight the most significant metabolic activities (**Figure 8**).

The enrichment analysis indicated that metabolic differentiation among cultivars is concentrated within specific secondary metabolic pathways. “Flavonoid biosynthesis,” “Phenylpropanoid biosynthesis,” and “Flavone and flavonol biosynthesis” emerged as the most significantly enriched core pathways across the comparisons. Specifically, “Phenylpropanoid biosynthesis”—the upstream backbone for flavonoid and phenolic synthesis—was significantly enriched in all comparison groups, suggesting that metabolic differentiation originates at the precursor level. In the DX vs RY comparison, KEGG enrichment analysis showed that ‘Biosynthesis of caffeic acid derivatives’ and ‘Biosynthesis of ferulic acid derivatives’ were among the top enriched pathways (**Figure 8A**). These pathways represent important branches of the broader phenylpropanoid biosynthesis pathway, suggesting activation of this upstream metabolic route in this comparison group. “Flavonoid biosynthesis” was significantly enriched in comparisons involving XGH, YL, and YS (e.g., XGH vs. RY, YL vs. XGH), while “Flavone and flavonol biosynthesis” was significantly enriched in all groups except DX vs. RY (**Figure 8**).

**Figure 9:**
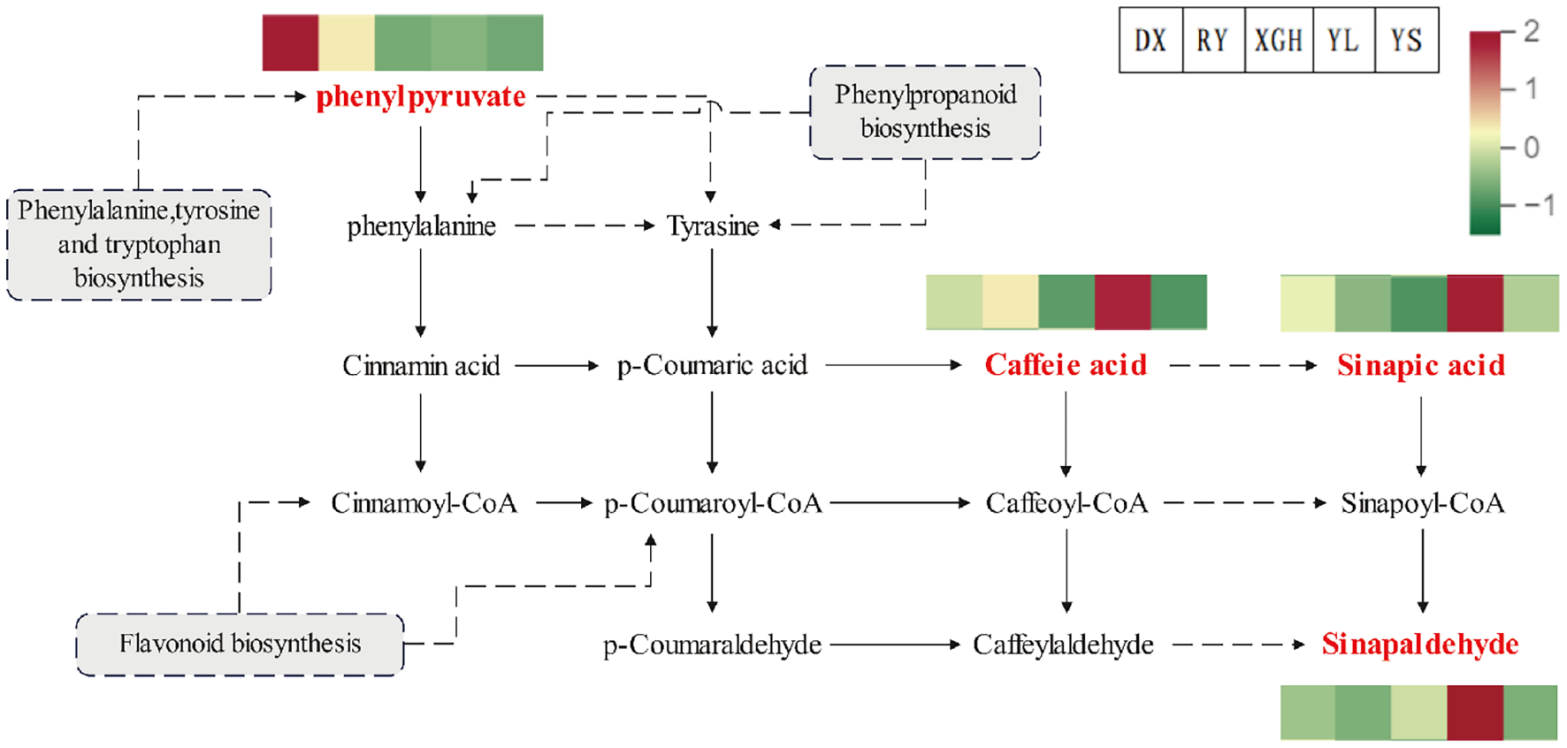
Overview of the phenylpropanoid biosynthesis pathway, showing the accumulation patterns of key precursors for flavonoid biosynthesis. Metabolites detected in this study are highlighted in bold red text; others represent undetected compounds. Small rectangles from left to right represent cultivars DX, RY, XGH, YL, and YS. Color within the rectangles shifts from green to red, indicating an increase in metabolite abundance from low to high. Solid arrows represent direct promotion, while dashed arrows indicate indirect promotion. (Figure description conventions same as **Figures 10**, **11**).

**Figure 10:**
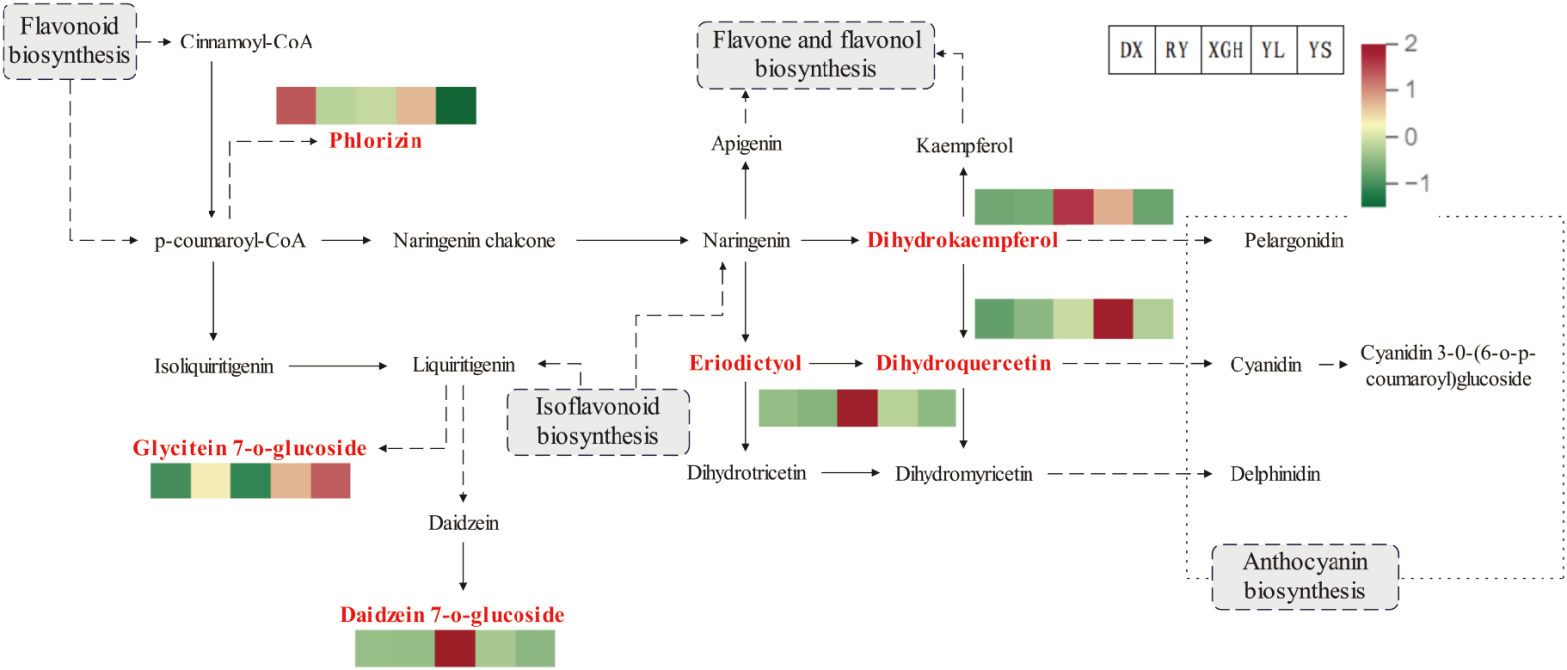
Overview of the flavonoid biosynthesis pathway, highlighting cultivar differences in mid-pathway metabolites.

**Figure 11:**
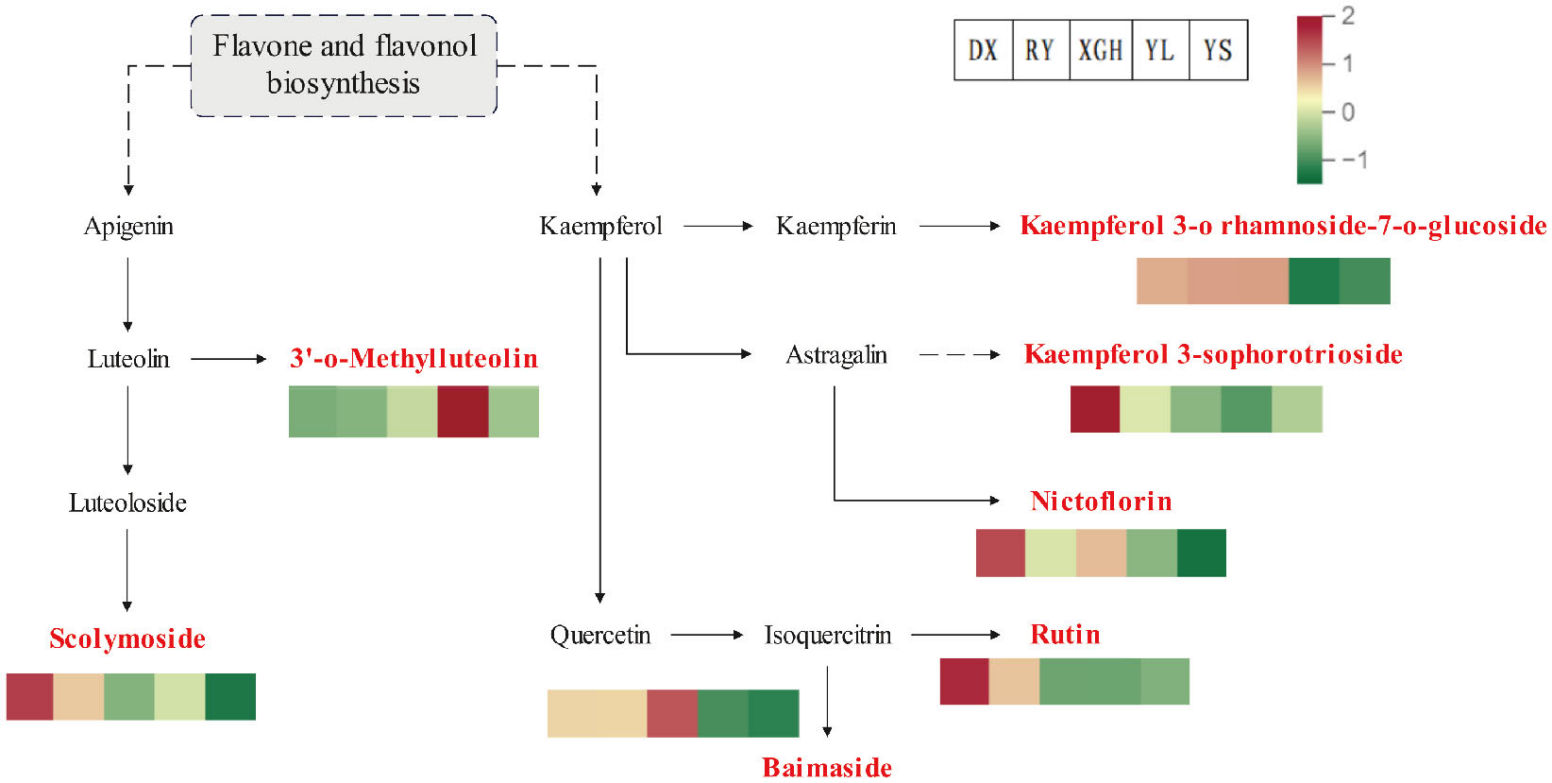
Overview of the flavone and flavonol biosynthesis pathway, presenting the final differentiation pattern of flavonoid metabolism at the product level.

We further reconstructed the metabolic maps for these three key pathways to visualize cultivar-specific accumulation patterns (**Figures 9**-**11**). In the Phenylpropanoid biosynthesis pathway (**Figure 9**), which proceeds from phenylalanine to hydroxycinnamic acids, YL exhibited a comprehensive up-regulation of Sinapic acid, Caffeic acid, and Sinapaldehyde compared to all other cultivars. In contrast, DX showed significant expression of Phenylpyruvate. In the Flavonoid biosynthesis pathway (**Figure 10**), which covers the conversion of chalcones to flavanones and flavanonols, YL was characterized by extremely high levels of Dihydroquercetin. XGH exhibited high expression of multiple intermediates, including Glycitin 7-O-glucoside, Eriodictyol, and Dihydrokaempferol. Phlorizin was abundantly accumulated in both DX and YL, while Glycitein 7-O-glucoside was highly expressed in YS. Finally, in the Flavone and flavonol biosynthesis pathway (**Figure 11**), distinct accumulation patterns of glycosylated products were observed. DX showed high levels of Scolymoside*, Rutin*, and Nictoflorin. XGH was characterized by the massive accumulation of Baimaside, while YL specifically accumulated 3’-O-Methylluteolin. Conversely, YS exhibited the lowest accumulation levels for the majority of flavonol glycosides in this pathway. These results map the specific biochemical bottlenecks and fluxes that drive the phenotypic diversity of *L. discolor* cultivars.

### 3.4 Targeted metabolomic analysis of flavonoids in five *L. discolor* cultivars

Based on the widely targeted metabolomics analysis, a metabolic differentiation network for flavonoid biosynthesis among *L. discolor* cultivars was constructed. This revealed that the accumulation of upstream precursors (e.g., Caffeic acid) and downstream glycosylation products (e.g., Dihydrokaempferol) exhibited high cultivar specificity. To validate the real content differences of key metabolites predicted by the pathway maps and to obtain absolute quantitative data for quality evaluation, targeted absolute quantification was performed, followed by variance analysis for inter-cultivar comparison.

**Figure 12:**
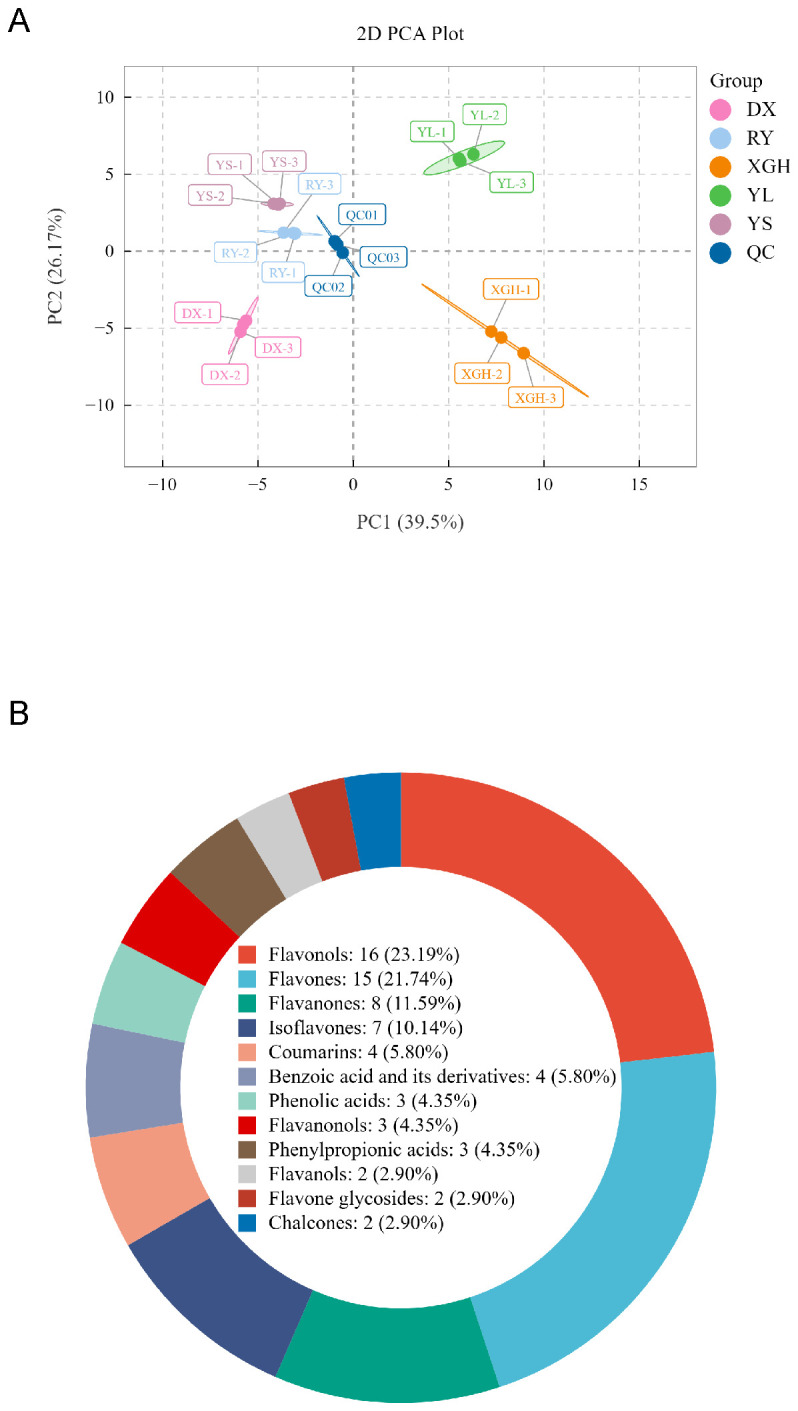
Quantitative flavonoid metabolome profile across the five *Ludisia discolor* cultivars. **(A)** Principal component analysis (PCA) score plot of samples. **(B)** Donut plot of identified flavonoid compound classification.

PCA of the targeted flavonoid metabolome showed a cumulative contribution rate of 65.67% for PC1 and PC2. Samples from the same cultivar clustered together, indicating high biological replicate consistency. QC samples also clustered tightly, confirming stable measurement conditions and high data quality (**Figure 12A**). A total of 69 substances were detected, comprising 16 flavonols, 15 flavones, 8 flavanones, 7 isoflavones, 4 coumarins, 4 benzoic acid derivatives, 3 phenolic acids, 3 phenylpropionic acids, 3 flavanonols, 2 flavanols, 2 flavone glycosides, and 2 chalcones (**Figure 12B**; details in **Supplementary Table 2**).

Eleven key flavonoid components selected from the predicted metabolic pathways were quantitatively analyzed to validate the widely targeted metabolomics findings. These 11 flavonoid compounds were selected because they are located at key nodes in significantly enriched pathways identified by KEGG enrichment analysis, including ‘phenylpropanoid biosynthesis’ and ‘flavonoid biosynthesis’ (**Figure 8**). They were significantly different across multiple cultivar comparisons, with VIP ≥ 1, FDR < 0.05, and |log_2_FC| ≥ 1. In addition, high-purity commercial standards were available for these compounds, ensuring accurate quantification. Some of these compounds have also been reported to possess antioxidant, anti-inflammatory, or other bioactivities in related medicinal plants. Results showed highly significant differences in the accumulation of these representative metabolites among cultivars, with accumulation patterns that strongly corroborated and refined the widely targeted data. Overall, XGH and YL held an absolute advantage in the accumulation of most key flavonoid components. Among the 11 quantified substances, more than 8 were significantly higher (*P* < 0.05) in XGH or YL compared to the other three cultivars, consistent with the distinct separation of their metabolic profiles in the PCA.

**Figure 13:**
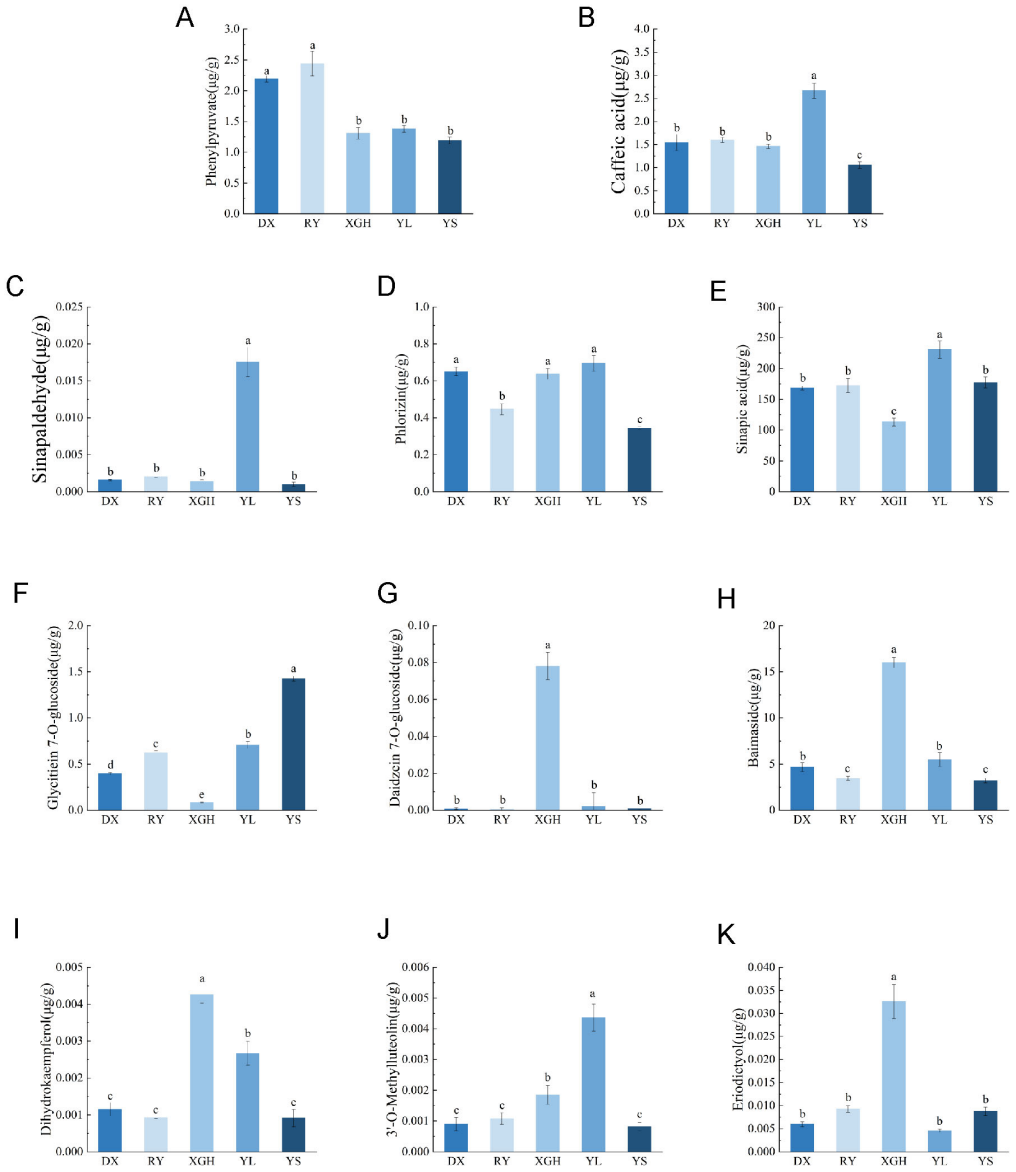
Absolute quantification analysis of 11 key flavonoid components in five *Ludisia discolor* cultivars. These 11 metabolites **(A–K)** were selected from key pathways in “flavonoid biosynthesis” based on widely targeted metabolomics and KEGG pathway enrichment analysis. Bar charts show the average content of each compound across cultivars (μg/g DW). Data are presented as mean ± standard error (n=3). One-way analysis of variance (ANOVA) followed by Tukey’s multiple comparison test was used for significance analysis. Different lowercase letters above bars indicate statistically significant differences (*P* < 0.05) in the content of the same metabolite among different cultivars.

Specifically, hierarchical differences in the metabolic pathways were clearly reflected in the quantitative data. Caffeic acid and Sinapic acid were highest in YL, significantly exceeding other cultivars (*P* < 0.05), consistent with the generalized up-regulation of hydroxycinnamic acids in YL observed in the pathway map (**Figure 9**). Phenylpyruvate was specifically accumulated in DX, potentially suggesting differences in phenylalanine metabolic flux branching or conversion efficiency. Dihydrokaempferol and Eriodictyol were highest in XGH. Phlorizin accumulated massively in both DX and YL, with the highest content in YL. The isoflavone glycosides Glycitin 7-O-glucoside and Daidzein 7-O-glucoside were primarily enriched in YS; although their relative content in the overall metabolic profile was low (**Figure 5B**), targeted quantification revealed YS’s specific accumulation advantage in the isoflavone branch. Regarding downstream flavonol glycosides, Baimaside was specifically highly expressed in XGH, perfectly matching the pathway prediction (**Figure 13**), while 3’-O-Methylluteolin content in YL was significantly higher than in all other cultivars.

## 4 Discussion

In this study, we employed metabolomics to systematically profile the metabolic landscapes of distinct *L. discolor* cultivars. The analysis revealed substantial heterogeneity in metabolite accumulation, identifying 802 differential metabolites predominantly comprising flavonoids, terpenoids, and lipids. Both PCA and OPLS-DA models demonstrated a clear segregation of the XGH and YL cultivars from the DX, RY, and YS groups, highlighting significant inter-cultivar metabolic differentiation. This phenomenon of cultivar-specific metabolic clustering aligns with recent findings in major crops such as soybean (Park et al., 2025), rice (He et al., 2025), and sorghum (Seo, 2025). Similarly, distinct metabolic signatures had been reported in pineapple, where K-means clustering effectively visualized cultivar-specific accumulation patterns of amino acids and coumarins (Zheng et al., 2025). These parallels strongly corroborate our finding that metabolic differentiation, particularly within secondary metabolism, is a fundamental characteristic of plant germplasm resources.

Importantly, metabolic differentiation serves as a chemical proxy for genetic diversity and medicinal potential. The significant variation in flavonoid distribution observed here mirrors findings in chrysanthemum, where total flavonoid content correlated directly with antioxidant activity and cultivar quality (Wang et al., 2024). Notably, we identified three ubiquitous differential metabolites across all comparison groups: Kaempferol-4'-O-glucoside*, 1-O-(3,4,5-Trimethoxybenzoyl)-β-D-Glucopyranoside, and sec-o-Glucosylhamaudol. These compounds accumulated prominently in YL and XGH, suggesting that they may serve as potential chemical markers for distinguishing major *Ludisia discolor* cultivars, particularly YL and XGH. These findings may provide a basis for rapid metabolic fingerprinting and cultivar authentication. Furthermore, we observed a potential link between metabolic profiles and morphological traits. In comparisons such as XGH vs. RY, the top 30 differential metabolites (by VIP score) were dominated by flavonoids like Kaempferol-4’-O-glucoside and Yuanhuanin, which were significantly up-regulated in XGH and YL. Interestingly, XGH, characterized by distinct golden-red veins, exhibited higher flavonoid glycoside levels than the pale-red veined RY. This accumulation may be linked to the functional role of flavonoids in photoprotection. The dark green leaves of XGH and YL suggest higher chlorophyll content and light capture efficiency, potentially necessitating an upregulated flavonoid pathway to cope with photo-oxidative pressure (Zhang et al., 2022; Ramabulana et al., 2016).

Flavonoid metabolism is a core secondary metabolic pathway governed by intricate regulatory mechanisms. KEGG pathway enrichment analysis confirmed that the differential metabolites in *L. discolor* were significantly enriched in “Phenylpropanoid biosynthesis,” “Flavonoid biosynthesis,” and “Flavone and flavonol biosynthesis.” This suggests that cultivar differentiation is driven by specific regulatory bottlenecks affecting the accumulation of key intermediates and end-products, a pattern also observed in lychee cultivars (Li and Wang, 2025). Our reconstruction of these biosynthetic pathways reveals distinct regulatory strategies. YL exhibited high accumulation of multiple hydroxycinnamic acids (e.g., Caffeic acid, Sinapic acid), suggesting a high metabolic flux through the upstream phenylpropanoid pathway that provides abundant precursors, consistent with reports of *L. discolor* being rich in phenolic substances (Ye et al., 2025). Conversely, XGH showed an accumulation advantage in dihydroflavonols (e.g., Dihydrokaempferol), indicating robust activity of enzymes such as flavonol synthase. Similar variations in key intermediates are considered primary drivers of chemical diversity in medicinal orchids like *Dendrobium* (Qiu et al., 2023).

The most pronounced differences appeared in downstream modifications. Analogous to findings in rapeseed (Shen et al., 2025), we observed a “shared core, divergent modification” pattern. XGH specifically accumulated Baimaside, YL was enriched in 3’-O-Methylluteolin, and YS showed a distinct profile of isoflavone glycosides. Glycosylation, methylation, and other modification reactions are critical processes that increase flavonoid diversity and functional plasticity. Glycosylation is commonly catalyzed by UDP-glycosyltransferases (UGTs), which can substantially enhance the water solubility and stability of flavonoids and may also influence their subcellular localization, transport, and biological activity (Ye et al., 2025). The distinct accumulation patterns of flavonol glycosides observed among *Ludisia discolor* cultivars, such as kaempferol-4’-O-glucoside, suggest that the expression levels or catalytic activities of UGT isoenzymes responsible for specific glycoside formation may differ among cultivars. Notably, although previous studies have identified genes involved in flavonoid biosynthesis in *L. discolor*, the functional specificity of the UGT family remains unclear. The metabolomic data generated in this study provides important phenotypic evidence and candidate targets for future investigation. However, differences in metabolite accumulation alone cannot distinguish whether these patterns arise from *UGT* allelic variation, transcriptional regulation, post-translational modification, or other mechanisms. Therefore, we present differential expressions or allelic variation of specific *UGT* genes as a testable hypothesis that may explain cultivar-specific differences in flavonoid glycoside accumulation. Future studies integrating transcriptomics, proteomics, and *in vitro* enzyme activity assays will be needed to identify the *UGT* genes responsible for the synthesis of these key glycosides and to clarify the underlying regulatory mechanisms.

Flavonol glycosides (e.g., quercetin-3-O-glucoside) are pivotal breeding targets due to their potent antioxidant, anti-inflammatory properties, and nutritional value. Our study identifies XGH and YL as superior germplasm resources significantly enriched in these compounds. This parallels breeding efforts in crops like black rice (He et al., 2025), *Torreya grandis* (Ma et al., 2025), blueberry (Xie et al., 2025), and chrysanthemum (Wang et al., 2024), where high flavonoid glycoside content is a marker for functional quality and antioxidant capacity (Lv et al., 2025). The capacity to accumulate flavonol glycosides is often dictated by UGT activity. In *Physalis alkekengi* (Yan et al., 2025) and *Platostoma palustre* (You et al., 2024), flavonol glycoside content correlates directly with *UGT* gene expression; similarly, *RsUGT74B1* expression drives flavonoid glycosylation in red radish (Cai et al., 2025). XGH and YL may possess favorable *UGT* alleles and could serve as promising candidates for molecular marker-assisted breeding, although this hypothesis requires further experimental validation.

Beyond medicinal quality, high flavonol glycoside content confers stress resistance. These compounds scavenge reactive oxygen species (ROS), aiding in cellular homeostasis under drought and other stresses (Cheng et al., 2025). Therefore, the high-flavonoid *L. discolor* cultivars identified here likely possess enhanced abiotic stress tolerance. Future breeding programs could leverage genome-wide selection combined with metabolomic screening to develop new cultivars that combine high medicinal yield with robust environmental resilience.

In summary, this study provides the first comprehensive metabolomic dissection of five *L. discolor* cultivars, demonstrating that XGH and YL possess distinct profiles significantly enriched in flavonol glycosides. Integrated pathway analysis confirmed phenylpropanoid and flavonoid biosynthesis as the core drivers of this divergence, while targeted quantification validated the superior accumulation of key bioactive compounds in XGH and YL. Additionally, three ubiquitous differential metabolites, including Kaempferol-4’-O-glucoside, were identified as potential biomarkers for cultivar authentication. These findings establish a critical foundation for precision germplasm evaluation and the breeding of high-quality medicinal cultivars, warranting future transcriptomic investigation into the genetic regulation of downstream flavonoid glycosylation. Although the widely targeted metabolomics data clearly indicate distinct glycosylation patterns, future transcriptomic or genomic studies are needed to confirm the underlying genetic or transcriptional regulatory mechanisms, such as allelic variation or differential expression of *UGT* genes.

## Data Availability

The original contributions presented in the study are included in the article/**Supplementary Material**, further inquiries can be directed to the corresponding author/s.

## Supplementary material

Supplementary Table 1 Statistics of differential metabolites across comparative groups.

Supplementary Table 2 Flavonoids detected by targeted quantitative metabolomics.
