# Supplementary material for: Integration of widely targeted and targeted metabolomics reveals flavonoid accumulation profiles across different cultivars of Ludisia discolor
Source: Front Plant Sci. 2026 Jul 7;17:1881198. doi: 10.3389/fpls.2026.1881198 (PMC13385630; doi:10.3389/fpls.2026.1881198)
Supplement: Supplementary Table 1 — Statistics of differential metabolites across comparative groups. [file Table1.docx]

**Table S1. Statistics of differential metabolites across comparative groups**

| *Class* | *DX vs. RY* | *XGH vs. RY* | *YL vs. RY* | *YS vs. RY* | *XGH vs. DX* | *YL vs. DX* | *YS vs. DX* | *YL vs. XGH* | *YS vs. XGH* | *YS vs. YL* |
| --- | --- | --- | --- | --- | --- | --- | --- | --- | --- | --- |
| Lipids | 89 | 129 | 87 | 72 | 76 | 151 | 94 | 192 | 141 | 71 |
| Terpenoids | 89 | 119 | 146 | 98 | 127 | 155 | 93 | 111 | 117 | 122 |
| Others | 100 | 113 | 139 | 104 | 119 | 155 | 86 | 141 | 130 | 141 |
| Flavonoids | 86 | 183 | 193 | 122 | 178 | 204 | 173 | 128 | 197 | 186 |
| Amino acids and derivatives | 93 | 104 | 111 | 61 | 137 | 123 | 76 | 86 | 105 | 100 |
| Alkaloids | 85 | 74 | 97 | 76 | 109 | 119 | 77 | 89 | 102 | 105 |
| Phenolic acids | 54 | 73 | 91 | 62 | 92 | 84 | 55 | 74 | 87 | 80 |
| Organic acids | 17 | 34 | 35 | 18 | 30 | 25 | 14 | 28 | 26 | 25 |
| Nucleotides and derivatives | 11 | 10 | 17 | 14 | 15 | 20 | 7 | 10 | 18 | 22 |
| Lignans and Coumarins | 30 | 29 | 40 | 34 | 37 | 38 | 23 | 31 | 37 | 32 |
| Quinones | 10 | 10 | 15 | 10 | 12 | 20 | 11 | 16 | 8 | 13 |
| Steroids | 4 | 7 | 7 | 1 | 2 | 11 | 3 | 10 | 7 | 5 |
